# An Error-Related Negativity Potential Investigation of Response Monitoring Function in Individuals with Internet Addiction Disorder

**DOI:** 10.3389/fnbeh.2013.00131

**Published:** 2013-09-25

**Authors:** Zhenhe Zhou, Cui Li, Hongmei Zhu

**Affiliations:** ^1^Department of Psychology, Wuxi Mental Health Center, Wuxi, China

**Keywords:** Internet addiction disorder, event-related potentials, error-related negativity, the modified Erikson flanker task, response monitoring function

## Abstract

Internet addiction disorder (IAD) is an impulse disorder or at least related to impulse control disorder. Deficits in executive functioning, including response monitoring, have been proposed as a hallmark feature of impulse control disorders. The error-related negativity (ERN) reflects individual’s ability to monitor behavior. Since IAD belongs to a compulsive-impulsive spectrum disorder, theoretically, it should present response monitoring functional deficit characteristics of some disorders, such as substance dependence, ADHD, or alcohol abuse, testing with an Erikson flanker task. Up to now, no studies on response monitoring functional deficit in IAD were reported. The purpose of the present study was to examine whether IAD displays response monitoring functional deficit characteristics in a modified Erikson flanker task. Twenty-three subjects were recruited as IAD group. Twenty-three matched age, gender, and education healthy persons were recruited as control group. All participants completed the modified Erikson flanker task while measured with event-related potentials. IAD group made more total error rates than did controls (*p* < 0.01); Reactive times for total error responses in IAD group were shorter than did controls (*p* < 0.01). The mean ERN amplitudes of total error response conditions at frontal electrode sites and at central electrode sites of IAD group were reduced compared with control group (all *p* < 0.01). These results revealed that IAD displays response monitoring functional deficit characteristics and shares ERN characteristics of compulsive-impulsive spectrum disorder.

## Introduction

With Internet’s rapid advance and social penetration, its negative effects have emerged prominently. Internet addiction disorder (IAD), also described as Pathological Internet use (PIU) or problematic Internet use, is defined as an individual’s inability to control his or her use of the Internet, which eventually causes psychological, social, school, and work difficulties or dysfunction in a person’s life (Young and Rogers, 1998; Davis, 2001). IAD has been increasingly recognized as a mental disorder. Many studies support the hypothesis that IAD is a new and often unrecognized clinical disorder that can cause relational, occupational, and social problems. Pathological gambling is compared to problematic Internet use because of overlapping diagnostic criteria. As computers are used with great frequency, detection, and diagnosis of Internet addiction is often difficult. Symptoms of a possible problem may be masked by legitimate use of the Internet (Griffiths, 2000; Block, 2008; Young, 2009; Weinstein and Lejoyeux, 2010). Recent estimates of its high prevalence in young people, combined with evidence that IAD is a maladaptive behavior with potentially serious occupational and mental health consequences, support the validity of the diagnosis (Ko et al., 2012). A recent study which analyzed Chinese college students who had been classified as computer addicts by the study designers and who used a computer around 10 h a day, 6 days a week, found reductions in the sizes of the dorsolateral prefrontal cortex, rostral anterior cingulate cortex, supplementary motor area, and parts of the cerebellum compared to students deemed “not addicted” by the designers. On the other hand, increases in the density of the right parahippocampal gyrus and a spot called the left posterior limb of the internal capsule were also found (Yuan et al., 2011). Another study which investigated the existence of differences in cortical thickness of the Orbitofrontal cortex (OFC) in adolescents with IAD displayed that subjects with IAD have significantly decreased cortical thickness in the right lateral OFC (Hong et al., 2013), which supports the view that the OFC alterations in adolescents with Internet addiction reflect a shared neurobiological marker of addiction-related disorders in general. However, there has been much disagreement in the planning for DSM-V about how to conceptualize this relatively new condition, or its core psychopathology (Holden, 2001).

However, there still has been the viewpoint that IAD is not a true addiction and may in fact be no more than a symptom of other, existing disorders. An overbroad description of addiction leaves open the possibility of every compensatory behavior being declared an addiction. For many individuals, overuse or inappropriate use of the Internet is a manifestation of their depression, anxiety, impulse control disorders, or pathological gambling. It is possible that a person could have a pathological relationship with a specific aspect of the Internet, such as bidding on online auctions, viewing pornography, online gaming, or online gambling (which is included under the existing Pathological Gambling), but that does not make the Internet medium itself addictive (Young and Rogers, 1998). Studies reported that IAD consists of at least three subtypes: excessive gaming, sexual preoccupations, and e-mail/text messaging. All of the subtypes share the common components, i.e., preoccupation, mood modification, excessive use, withdrawal, tolerance, and functional impairment (Block, 2008). By using the Diagnostic and Statistical Manual of Mental Disorders (Fourth Edition, DSM-IV) criteria, some authors suggest IAD is an impulse disorder or at least related to impulse control disorder (Brard and Wolf, 2001; Shaw and Black, 2008).

Components of impulsivity include attention, suppressing responses, poor evaluation of consequences, and/or an inability to forgo immediate small rewards in favor of greater delayed rewards. Impulsivity can be conceptualized more broadly as dysregulated behavior. Dysregulated behavior conveys the complex interplay of factors underlying the breakdown in behavioral regulation that the construct of impulsivity implies, such as poorly planned, unreflective, reckless, abrupt, under-controlled, or inappropriate behavior that leads to negative outcomes (Finn et al., 1999). A study which investigated the independence of measures of impulsivity and their association with hazardous drinking indicated that multiple components of impulsivity and automatic alcohol approach tendencies explain unique variance in hazardous drinking (Christiansen et al., 2012). Despite the impulsivity is not a monolithic trait, but a collection of distinct behavioral tendencies, however, some of which are major factors and vulnerability markers for addiction (Lawrence et al., 2009a,b). A study that investigated deficient inhibitory control in individuals with IAD using a visual go/no-go task by event-related potentials (ERPs) indicated individuals with IAD were more impulsive than controls and shared neuropsychological and ERPs characteristics of compulsive-impulsive spectrum disorder, which supports that IAD is an impulse disorder, or at least related to impulse control disorder (Zhou et al., 2010). Response monitoring is one of several cognitive processes subsumed under the umbrella term of executive functions, a class of processes thought to underlie flexible goal-directed behavior and attributed generally to the functioning of a network of inter-related neural regions including the prefrontal cortex, anterior cingulate, basal ganglia, and striatum (Shallice et al., 1991; Stuss et al., 1995). Response monitoring refers generally to the ability to monitor one’s own actions and progress toward a predefined goal and thus is essential for the successful execution of goal-directed behaviors. Specifically, when one is performing an action an internally generated monitoring system compares a representation of the correct or intended action with a representation of the actual response. If no discrepancy is detected current actions continue but if a discrepancy is detected, remedial actions are initiated (McKee et al., 1998; Coles et al., 2001).

The ERPs reflect the rapidly changing electrical activity associated with a cognitive event in relatively large synaptic fields containing tens of millions of neurons. When participants make errors in these speeded response tasks (such as, an Erikson flanker task), an ERP component, the error-related negativity (ERN), and presents as a negative detection approximately 50–100 ms following the erroneous response. Previous studies dedicated that ERN reflects error-related brain activity, namely, it reflects individual’s ability to monitor behavior (Falkenstein et al., 1991, 2000). Deficits in executive functioning, including response monitoring, have been proposed as a hallmark feature of impulse control disorders, including attention deficit disorder, obsessive compulsive disorder, and substance dependence. For example, a study which determined whether cocaine-dependent persons have error-processing deficits as measured using ERN showed when performing an Eriksen flanker task, Cocaine-addicted patients showed reduced ERN as compared to a control group. On the behavioral level, patients showed reduced post-error accuracy improvement. The findings reveal that cocaine addiction is associated with reduced error processing and impaired behavioral correction of errors after an error is made. These deficits may be associated with a compromised dopamine system (Franken et al., 2007). Another study measured the response-locked ERP during a flanker task with performance-based monetarily rewarding and punishing trials in 37 undergraduate students separated into high- and low-impulsive groups based on a median split on self-reported Barrett Impulsiveness Scale. The high-impulsive group had a smaller medial frontal ERN on punishment trials than the low-impulsive group. The medial prefrontal neural system of behavior monitoring, indexed by the ERN, appears less sensitive to punishment signals in normal impulsivity. This reduced punishment sensitivity in impulsivity, a personality variation associated with several mental and personality disorders including ADHD and substance abuse may be related to the tendency to select short-term rewards despite potential long-term negative consequences in these individuals (Potts et al., 2006). A previous study investigated whether smokers showed initial error processing deficits, as measured with ERN, when exposed to smoking cues. ERN was measured during a modified Erikson flanker task in both smokers and non-smoking controls. Results showed smokers showed reduced ERN amplitudes after making an error, accompanied by diminished post-error slowing of reaction times (RTs). These results suggest that initial error processing attributed to an error is affected in smokers during smoking cue exposure. Furthermore, individual variation in impulsivity and nicotine dependence was associated with reduced ERN amplitudes (Luijten et al., 2011). Since IAD belongs to a compulsive-impulsive spectrum disorder, theoretically, it should present response monitoring functional deficit characteristics of some disorders, such as substance dependence, ADHD, or alcohol abuse, testing with an Erikson flanker task. Up to now, no studies on response monitoring functional deficit in IAD were reported. In this study, participants’ behavioral responses and ERPs were recorded while they performed a modified Erikson flanker task. The ERN was suitable to examine the neural processes involved with response monitoring function. The purpose of the present study was to examine whether IAD displays response monitoring functional deficit characteristics in a modified Erikson flanker task.

## Materials and Methods

### Time and setting

The experiment was completed in the Department of psychology at Wuxi Mental Health Center, China, from May 2009 to March 2012.

### Diagnostic approaches and participants

The criteria of IAD group included: (a) met the criteria of the modified Diagnostic Questionnaire for Internet Addiction (YDQ) (see Appendix) (Brard and Wolf, 2001), i.e., subjects who answered “yes” to questions 1 through 5 and at least any one of the remaining three questions were classified as suffering from IAD; (b) whose age were more than 18 years old; (c) did not meet criteria of any DSM-IV axis I disorder or personality disorders by administering a structured clinical interview (Chinese version); (d) were not smokers; and (e) had not a diagnosis of alcohol or substance dependence, neurological disorders, all kinds of head injury or systemic disease that might affect the central nervous system.

The duration of the disorder was estimated via a retrospective diagnosis. We asked the subjects to recall their life-style when they were initially addicted to the Internet. To guarantee that they were suffering from Internet addiction, we retested them with the criteria of the modified YDQ. We also confirmed the reliability of these self-reports from the IAD subjects by talking with their parents via telephone. The IAD subjects spent 11.01 ± 1.52 h/day on online activities (including pornography, gaming, virtual society, Internet social interaction, and obtaining information). The days of Internet use per week was 6.41 ± 0.6. We also verified this information from the roommates and classmates of the IAD subjects that they often insisted being on the Internet late at night, disrupting others’ lives despite the consequences. Subjects were recruited from IAD Therapeutic Department of Wuxi Mental Health Center. They have regulated sleep patterns and did not ingest large quantities of caffeinated and energetic drinks by medical staffs’ management.

Twenty-three subjects were recruited as IAD group. The controls were recruited from citizens lived in Wuxi city, Jiangsu Province, China through local advertisement. Controls were excluded from the study if they were smokers; or had a diagnosis of alcohol or substance dependence, neurological disorders, all kinds of head injury or systemic disease that might affect the central nervous system. Twenty-three matched age, gender, and education healthy persons were recruited as control group. According to a previous IAD study (Ko et al., 2009), we chose healthy controls who spent less than 2 h/day on the Internet. The controls were also tested with the YDQ criteria modified by Beard and Wolf to ensure they were not suffering from IAD. All participants were Chinese. All participants underwent a clinical assessment by a psychiatrist to collect information on medication, socio-demographic data, and to confirm/exclude an IAD diagnosis. Handedness was assessed using the Annett handedness scale (Annett, 1970). Ratings on this scale were recorded into the following definitions of handedness: Annett score (1) = right, (2–7) = mixed, (8) = left. In this study, we gave all participants a written informed consent to participate and all were paid. The protocol for the research project was approved by the Ethics Committee of Nanjing Medical University, China.

### Tasks and procedure

#### The modified Erikson flanker task

E-Prime software 2.0 (Psychology Software Tools Inc., Sharpsburg, NC, USA) was used for the experimental procedure. The modified Erikson flanker task, adapted from Heather et al. (2006), after acquiring 6 min of eyes open and eyes closed resting electroencephalography (EEG), a modified version of the Eriksen flanker task was administered. In this task participants are required to indicate the direction of a central target in an array of five stimuli on compatible trials (< < < < < or > > > > >) or on incompatible trials (< < > < < or > > < > >), in which the target stimulus faces in the opposite direction from all other stimuli in the array. Correct performance required the participants to press the button on the keypad that corresponded to the direction of the center arrowhead. Participants were seated approximately 70 cm from a computer monitor holding a small box with two buttons on his/her lap. A small fixation mark (a red dot) remained in the center of the monitor throughout the task, with the stimulus arrays presented just above the fixation mark. Participants completed a shortened block of practice trials and timing trials prior to cap placement. Participants who committed eight errors or less during the 50 timing trials, were administered 3 blocks of 96 (288) data collection trials consisting of a 200 ms warning cue (an asterisk), a 300 ms delay, and one of the four target displays lasting for 200 ms. They then had 800 ms to make their response. Participants who committed more than eight errors during the timing trials, were administered 3 blocks of 96 (288) data collection trials consisting of a 200 ms warning cue, a 300 ms delay, and one of four target displays lasting for 250 ms, with 1100 ms to make their response. The order of compatible and incompatible trials was counterbalanced so that the probability of each target display was 0.25 across a block of trials. Each task block lasted approximately 7 min, for a total of 21 min of testing.

#### Behavioral analysis

According to previous study (Heather et al., 2006), RT was recorded for every trial and mean RTs were computed for total error responses, including compatible and incompatible trials. RT measures began with the presentation of the target display and ended when a button press was detected or when the trial ended, whichever came earlier. Error rates were computed for total trails, including compatible and incompatible trials.

#### Electrophysiological recordings

Depending on the findings and reports of studies that the ERN is typically measured at midline frontal or central sites (Doreen and Greg, 2008), according to the 10/20 International System, EEG was recorded with the Stellate Harmonie EEG device (Physiotec Electronics Ltd., Canada) using Electro-Cap Electrode System (ECITM Electro-Caps, Electro-cap International, INL, USA) from F3, Fz, F4, C3, Cz, C4, A1, and A2. Combined ear electrodes served as a reference and the ground electrode was attached to the forehead. Eye movement artifacts were monitored by recording vertical and horizontal electro oculogram (EOG) from electrodes placed above and below the right eye and at the left outer canthus. Electrode impedance was kept below 5 kΩ. System band pass was 0.1–30 Hz and digitalized continuously at a sampling rate of 250 Hz. The EEG activity was recorded only during the recording phase not the practice phase.

#### ERP analysis

The Brain Electrical Source Analysis program (BESA 5.2.0 Software, Graefelfing, Germany) was used to perform data analysis. Epochs were constructed that consisted of a 100 ms pre-stimulus baseline and a 1000 ms post-stimulus interval. All epochs with amplitudes exceeding ±75 μV at any electrode were excluded automatically. Epochs were averaged offline for each subject and stimulus type and digitally filtered with a low-pass filter of 15 Hz (24 dB down). Measurement latency windows were determined based on visual inspection of the individual data and grand-averaged data of all subject. Inspection of the grand-average waveforms indicated that ERN component, the peak negativity within a 50–100 ms latency window, was used for analysis.

#### Statistical analysis

Data collected were analyzed with SPSS 10.0 statistical software (SPSS, Chicago, IL, USA). Comparisons of RTs and error rates between IAD group and control group were done using independent-sample *t*-tests. Separate repeated-measures analysis of variance (ANOVA) was performed for ERPs from frontal (F3, Fz, and F4) and central (C3, Cz, and C4) electrode sites for ERN amplitudes. All F ratios associated with repeated-measures factors were assessed using degrees of freedom corrected with Greenhouse–Geisser procedure for controlling Type I error. Least square difference (LSD) tests were performed as *post hoc* analyses if indicated. Alpha values of 0.05 were considered significant throughout.

## Results

### Demographic characteristics of participants

The demographic characteristics of all subjects are detailed in Table 1. There were no differences in sex ratio, mean age, mean education years, and handedness between the two groups (*p* > 0.05).

**Table 1:** **Demographic characteristics of the sample**.

	IAD group	Control group
Sex ratio (M/F)	23 (17:6)	23 (17:6)
Mean age (SD)	25 (6)	25 (6)
Age range	18–36	18–36
Mean education years (SD)	9 (4)	9 (4)
**HANDEDNESS**		
R/M/L	13/7/3	14/6/3
(% R/M/L)	(57/30/13%)	(61/26/13%)

M, male; F, female; SD, standard deviation; R, right; M, mixed; L, left.

The demographic characteristics of the sample are detailed in Table 1.

### Assessment of behavioral outcome

By independent-sample *t*-tests, IAD group made more total error rates [(19.8 ± 3.45)%] than did controls [(13.1 ± 2.67)%] (*t* = 3.238, *p* < 0.01); RTs for total error responses in IAD group [(440 ± 21) ms] were shorter than did controls [(495 ± 18) ms] (*t* = −2.963, *p* < 0.01).

### Comparisons of ERN amplitudes of total error response conditions between IAD group and control group

Error-related negativity amplitudes of total error response conditions at frontal electrode sites and *central electrode sites* showed as Table 2.

**Table 2:** **Error-related negativity amplitudes [(***μ***V) (mean ***±*** SD)] of total error response conditions in IAD group and control group**.

Group	F3	Fz	F4	C3	Cz	C4
Control	9.2 ± 1.6	9.1 ± 1.3	8.5 ± 1.9	8.9 ± 2.0	9.0 ± 2.3	8.7 ± 1.7
IAD	1.3 ± 0.3	1.1 ± 0.6	1.9 ± 0.8	1.2 ± 0.5	1.4 ± 0.7	1.0 ± 0.7

#### ERN amplitudes of total error response conditions at frontal electrode sites

**Figure 1:**
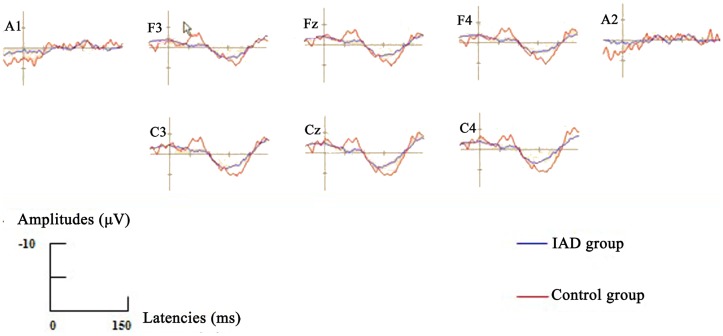
**Grand-averaged ERPs waveforms for error (ERN, designated with cursor at F3) were elicited by the modified Erikson flanker task for IAD group (blue lines) and Control group (red lines)**. The ERN components were presented within a 50–100 ms latency window.

A repeated measure ANOVA with frontal electrode sites (F3, Fz, and F4) and group (IAD vs. control) as within-subject factors revealed a significant group, frontal electrode sites and group × frontal electrode sites main effect for ERN amplitudes (for group: *F* = 768, df = 1, *p* = 0.000; for frontal electrode sites: *F* = 615, df = 2, *p* = 0.000; for group × frontal electrode sites: *F* = 516, df = 2, *p* = 0.000). LSD tests were performed as *post hoc* analyses and demonstrated significant differences between ERN amplitudes at frontal electrode sites of IAD group and those at control group (all *p* = 0.000). ERN amplitudes were lower than those at control group (Figure 1).

#### ERN amplitudes of total error response conditions at central electrode sites

A repeated measure ANOVA with frontal electrode sites (C3, Cz, and C4) and group (IAD vs. control) as within-subject factors revealed a significant group, central electrode sites and group × central electrode sites main effect for ERN amplitudes (for group: *F* = 862, df = 1, *p* = 0.000; for central electrode sites: *F* = 599, df = 2, *p* = 0.000; for group × central electrode sites: *F* = 483, df = 2, *p* = 0.000). LSD tests were performed as *post hoc* analyses and demonstrated significant differences between ERN amplitudes at central electrode sites of IAD group and those at control group (all *p* = 0.000). ERN amplitudes were lower than those at control group (Figure 1).

## Discussion

This study is the first to employ the modified Erikson flanker task to investigate response monitoring functional deficit characteristics in IAD with ERN. Our trail results showed that when performing the modified Erikson flanker task, IAD group made more total error rates than did controls, and reactive times for total error responses in IAD group were shorter than did controls. Consistent with previous study (Cao and Su, 2007), individuals with IAD were more impulsive than controls. A pervious small sample size study on psychiatric features of individuals with problematic Internet use showed that all subjects’ problematic Internet use met DSM-IV criteria for an impulse control disorder not otherwise specified, and concluded that problematic Internet use may be associated with subjective distress, functional impairment, and Axis I psychiatric disorders (Shapira et al., 2000). Another study used a questionnaire survey on Internet addicted behavior displayed a high prevalence of features of impulse control disorders, such that presented a great urge to be “online” if they are disconnected; felt the world is an empty and dull space without Internet; had daytime fantasies about Internet use; became very nervous if the Internet connection was slow; displayed depressive mood and of feeling guilty after a longer use of the web; had aggressive behaviors if they were interrupted by others using the Internet (Treuer et al., 2001). Above mentioned two studies suggested that IAD is a new subtype of impulse control disorder. Within neuropsychology and cognitive neuroscience, impulsivity is often equated with the term “disinhibition,” referring to the idea that top-down control mechanisms ordinarily suppress automatic or reward-driven responses that are not appropriate to the current demands (Aron, 2007). These inhibitory control mechanisms may be disrupted following pathological gambling, drug addiction, ADHD, or alcohol abuse, resulting in a predisposition toward impulsive acts. Defined in this way, impulsivity has relevance to IAD.

A hallmark personality characteristic in substance abuse is impulsivity (Moeller et al., 2001). Impulsivity is related to increased sensitivity to reward and decreased sensitivity to punishment; studies show that individuals who score high on impulsivity scales have decreased ERN amplitudes in response to errors (Ruchsow et al., 2005; Potts et al., 2006). Our study showed that ERN amplitudes of total error response conditions at frontal electrode sites and at central electrode sites of IAD group were reduced compared with control group. The ERN variations in IAD are similar to some impulse control disorders, such as attention deficit disorder, obsessive compulsive disorder, and substance dependence. Several theories and computational models have been developed regarding the functional significance of the ERN. Some suggest that the ERN reflects the error-detection process (Nieuwenhuis et al., 2001), an error signal at the remedial action system (Holroyd and Coles, 2002), the conflict-detection process (Yeung et al., 2004), or a more emotionally or motivationally relevant response to errors (Gehring and Willoughby, 2002). Since previous studies have shown that ERN reflects error-related brain activity, namely, it reflects individual’s ability to monitor behavior (Falkenstein et al., 1991, 2000). This study results suggested that individuals with IAD presented deficits in executive functioning, including response monitoring. The results of this study clearly demonstrate individuals with IAD were more impulsive than controls and shared neuropsychological and ERN characteristics of some disorders, such as pathological gambling, substance abuse, testing with the modified Erikson flanker task.

This study had several limitations that should be considered. Firstly, we did not employ Barratt Impulsiveness Scale 11 to measures of impulsivity; therefore, we did not provide the correlations between impulsiveness and ERN amplitudes. We would like to employ the method to improve the conclusion in the future research. Secondly, because of the small sample size our research results are preliminary. Further studies with larger sample sizes are needed to replicate our findings. Thirdly, because the IAD sample portrays a number of features that could account for the effects that showed the existence of some degree of disinhibition and response monitoring functional deficit, the differences between IAD subjects and controls could be attributed to a myriad of factors. The further research that employs a very careful selection and assessment of the samples on IAD should be done in the future. Finally, this study used YDQ scores of higher than 6 as an indicator of IAD. Although this questionnaire is a frequently used instrument for assessing IAD, its validity as a diagnostic instrument has been questioned (Beard, 2005). Future studies may utilize other measures of assessing diagnostic criteria or severity for IAD of Internet addiction problems to assess the relationship between response monitoring functional deficit and IAD.

## Conflict of Interest Statement

The authors declare that the research was conducted in the absence of any commercial or financial relationships that could be construed as a potential conflict of interest.

## Appendix

### Diagnostic questionnaire for internet addiction (YDQ)

- Do you feel preoccupied with the Internet (think about previous online activity or anticipate next online session)?
- Do you feel the need to use the Internet with increasing amounts of time in order to achieve satisfaction?
- Have you repeatedly made unsuccessful efforts to control, cut back, or stop Internet use?
- Do you feel restless, moody, depressed, or irritable when attempting to cut down or stop Internet use?
- Do you stay online longer than originally intended?
- Have you jeopardized or risked the loss of significant relationship, job, educational, or career opportunity because of the Internet?
- Have you lied to family members, therapist, or others to conceal the extent of involvement with the Internet?
- Do you use the Internet as a way of escaping from problems or of relieving a dysphoric mood (e.g., feelings of helplessness, guilt, anxiety, or depression)?
